# A Review of Commonly Used Methodologies for Assessing the Antibacterial Activity of Honey and Honey Products

**DOI:** 10.3390/antibiotics11070975

**Published:** 2022-07-20

**Authors:** Md Lokman Hossain, Lee Yong Lim, Katherine Hammer, Dhanushka Hettiarachchi, Cornelia Locher

**Affiliations:** 1 Division of Pharmacy, School of Allied Health, University of Western Australia, Crawley 6009, Australia; mdlokman.hossain@research.uwa.edu.au (M.L.H.); lee.lim@uwa.edu.au (L.Y.L.); dhanushka.hettiarachchi@outlook.com (D.H.); 2 School of Biomedical Sciences, University of Western Australia, Crawley 6009, Australia; katherine.hammer@uwa.edu.au; 3 CRC for Honey Bee Products, University of Western Australia, Crawley 6009, Australia

**Keywords:** honey, honey-based formulation, antimicrobial activity, apitherapy

## Abstract

Honey, a naturally sweet and viscous substance is mainly produced by honeybees (*Apis mellifera*) from flower nectar. Honey exerts a plethora of biological and pharmacological activities, namely, antioxidant, antimicrobial and anti-inflammatory activity, because of the presence of an extensive variety of bioactive compounds. The antibacterial activity is one of the most reported biological properties, with many studies demonstrating that honey is active against clinically important pathogens. As a result, beside honey’s widespread utilization as a common food and flavouring agent, honey is an attractive natural antimicrobial agent. However, the use of neat honey for therapeutic purposes poses some problems, for instance, its stickiness may hamper its appeal to consumers and health care professionals, and the maintenance of an adequate therapeutic concentration over a sufficient timeframe may be challenging due to honey liquidity and leakage. It has motivated researchers to integrate honey into diverse formulations, for example, hydrogels, dressings, ointments, pastes and lozenges. The antibacterial activity of these formulations should be scientifically determined to underscore claims of effectiveness. Some researchers have made efforts to adapt the disc carrier and suspension test to assess the antimicrobial activity of topical products (e.g., silver-based wound dressings). However, there is currently no established and validated method for determining the in vitro antimicrobial potential of natural product-based formulations, including those containing honey as the active principle. Against the backdrop of a brief discussion of the parameters that contribute to its antibacterial activity, this review provides an outline of the methods currently used for investigating the antibacterial activity of neat honey and discusses their limitations for application to honey-based formulations.

## 1. Introduction

### 1.1. Chemistry and Bioactivity of Honey

Honey is a supersaturated sugary and flavourful natural product of great nutritional value. It is also used for its perceived positive impacts on human health, in particular for its antioxidant, antimicrobial and anti-inflammatory and antimicrobial properties as well as its wound and (sun) burn remedial effects [1]. Honey bees make honey from plant nectars, and a similar product, honeydew, is produced from plant discharges and excretions. The chemical constituents of honey vary somewhat and are mainly influenced by its floral source and geographical origin [2,3], with climatic aspects along with processing conditions also influencing its composition and biological effects [4]. Chemically, honey is a mixture of mainly sugars (about 80%) and water (approximately 17%), alongside many minor components (approximately 3% in total), for instance, proteins, amino acids, organic acids, vitamins, minerals, polyphenols and volatile compounds [4,5,6,7,8]. The predominant sugars in honey are fructose and glucose, with very small amounts of other mono-, di- or oligosaccharides (maltose, sucrose, nigerose, isomaltose, turanose and maltulose) also identified [7,9]. The inherent acidic (pH 3–5) nature of honey predominantly comes from the breakdown of glucose into gluconic acid through enzymatic action of glucose oxidase [9,10]. Honey’s physicochemical characteristics, namely its taste, color, viscosity and solubility, are significantly influenced by its moisture content (about 17% of total). The water content is also responsible for the potential microbial contamination of honeys [10]. The minor fraction of ‘other’ constituents is considered important not only in terms of its physicochemical characteristics but also for the honey’s bioactivities [11]. These ‘other’ honey components are commonly referred to as non-sugar constituents and comprise of flavonoids (e.g., myricetin, kaempferol, kaempferol, quercetin, isorhamnetin, pinobanksin, rutin, galangin, genkwanin, luteolin, apigenin, tricetin, chrysin, pinocembrin, pinostrobin), phenolic acids (e.g., methyl syringate, gallic acid, ellagic acid, protocatechuic acid, syringic acid, benzoic acid, 4-hydroxybenzoic acid, chlorogenic acid, vanillic acid, caffeic acid, *p*-coumaric acid, ferulic acid, homogentisic acid, phenylacetic acid) and other organic acids, proteins (enzymes), amino acids, minerals (Ca^2+^, Cu^2+^, Fe^2+^, Mg^2+^, Mn^2+^, P^3+^, K^+^, Na^+^, Zn^2+^), vitamins (specifically vitamin C, vitamin B_6_, thiamine, niacin, riboflavin and pantothenic acid), pigments and numerous other compounds [7,8,9,10,11]. Several enzymes, specifically invertase, amylase, catalase and glucose oxidase, are also found in honey, and proline is a key amino acid, responsible for about half of honey’s total free amino acids [10].

Honey has been extensively used as a therapeutic agent for the treatment of numerous diseases [12,13]. It is highly valued and plays a significant role in a novel branch of alternative medicine, termed ‘apitherapy’, which emphases the medicinal use of honey as well as other bee and hive products [14,15]. Honey demonstrates beneficial effects in many physiological systems, for example, the cardiovascular, nervous, respiratory and gastrointestinal systems [16]. Honey might exert antimicrobial and/or antioxidant activities due to its high osmolarity, acidity, generation of H_2_O_2_ and NO on exposure to water, as well as the presence of so-called non-peroxide factors like methylglyoxal (MGO) [16]. Furthermore, phenolic compounds, organic acids, enzymes (e.g., diastase, glucose oxidase, and invertase), minerals (e.g., potassium, iron, zinc) and other minor constituents also have potential anti-parasitic, and antidiabetic activities [12].

The antibacterial activity of honey has been associated with the generation of hydrogen peroxide in the case of so-called ‘peroxide honeys’ [17,18,19] or the presence of methylglyoxal (MGO) in so-called ‘non-peroxide honeys’ [20] as well as the activity of bee defensin-1 and other bee-related enzymes alongside high osmolarity and a low pH. Flavonoids and phenolic substances also contribute to the antibacterial effect of both peroxide and non-peroxide honeys [19,20] in addition to their well-documented antioxidant effects [18,21].

Honey has been found to provide beneficial effects to different stages of wound healing (i.e., haemostasis, inflammation, remodeling) and thus to influence positively the natural physiology of wound healing, particularly by reducing oedema and wound exudate [17]. Honey also improves wound healing through the promotion of collagen synthesis, growth of new blood vessels, autolytic stimulation of the growth of fibroblasts cells, epithelial cells and granulation tissue and prevention of scar tissue and keloid formation [18]. Additionally, the antibacterial and antioxidant activities of honey also contribute to its wound remedial effects. These benefits provide the rationale for honey to be used as a potential antibacterial (and anti-inflammatory) agent in a range of medicinal products.

### 1.2. Honey Based Formulations

Compared to neat honey, which is a sticky and viscous liquid, honey-based medicinal products might be more convenient to use, and they also offer a more targeted therapeutical use. So far, only a few types of honey have been developed into medicinal formulations combined with additional materials, for instance alginate, collagen, gelatine, starch, cellulose [7,9,15]. Some honey-based products, such as gels, dressings, ointments have been approved by the US Food and Drug Administration (FDA) [7]. The majority of honeys incorporated into these formulations are obtained from the tree genus *Leptospermum*, which is native to Australia and New Zealand, and these honeys are usually referred to as Manuka honeys. Most of the commercial honey-based medicinal formulations have common topical uses, such as in the treatment of various wounds (e.g., minor abrasions, lacerations, cuts, scalds and burns, diabetic foot ulcers, leg ulcers, pressure ulcers/sores, traumatic and surgical wounds) [7].

### 1.3. Preclinical Evaluation of Antibacterial Activity

As with every medicinal formulation, the preclinical demonstration of bioactivity is paramount and an appropriate in vitro method should be used for the determination of antibacterial activity of the honey products. At present, however, diverse methodologies have been reported in the literature without a clear consensus for a standardised method for the assessment of the antibacterial activity of honey and honey-based medicinal formulations. One reason might be the inherent complexity of the different factors that contribute to a honey’s antibacterial properties, and the additional factors that have to be considered when the honey is formulated with other materials into the medicinal product. This review will first discuss these factors in details before current techniques for the assessment of the antibacterial activity of neat honeys and their limitations for the analysis of honey-based formulations are critically reviewed.

## 2. Factors Contributing to the Antibacterial Activity of Honey

**Figure 1:**
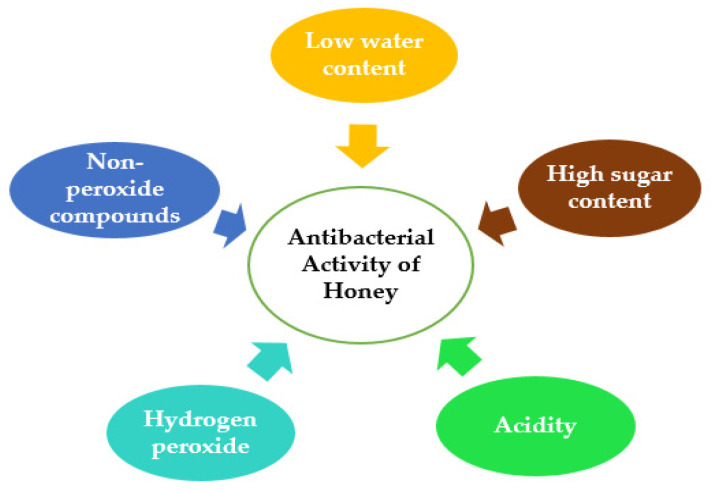
Schematic diagram presenting the parameters contribute to the antimicrobial activity of honey.

The antibacterial activity of honey is attributed and influenced by various properties of honey, such as low water content, high viscosity, acidity, hydrogen peroxide content, non-peroxide components, particularly the presence of MGO [22,23], peptides, non-peroxidase glycopeptides and proteins (Figure 1). These are all, to varying degrees, prominent aspects of honey’s antibacterial action [23].

### 2.1. Low Water Content

Water activity refers to the unbound water molecules in a sample with a proportional relationship between the unbound water and bacterial contamination. The normal range of water activity (a_w_) of honey is 0.562 to 0.62, which is less than the range confirmed as totally inhibiting the growth of bacteria (0.94–0.99) [23]. Thus, neat honey provides very low water content to facilitate the growth of microorganisms.

### 2.2. High Sugar Content

Honey is characterized by high sugar concentrations (70–80%) which can induce osmosis when administered to living cells. The osmosis is one of the vital features of honey to exert antibacterial activity against clinically important pathogenic bacteria [23]. The level of bacterial inhibition depends on the aqueous concentration of the honey along with the types of bacteria being considered [23]. Undiluted honey with its high sugar concentration is strongly hypertonic and can inhibit bacterial growth completely as its osmotic pressure will cause the transport of water out of the bacterial cells which ultimately result in cell death [24]. Neat honey when applied to infection sites, in particular exudating sites where the honey can be significantly diluted by body fluids, may lose its antibacterial action or be active only against certain bacterial species [25]. Furthermore, studies have shown that an ‘artificial’ honey solution (prepared with mono- and disaccharides at the same concentration as those found in honey) has little or no antibacterial activity against most bacteria [24]. This implies that factors other than sugars will also need to be considered when assessing the overall antibacterial effect of honey.

### 2.3. Acidity

The acidity of honey (pH 3.2–4.5) is significantly lower than the favourable pH (6.5–7.5) for the growth of most bacteria [25]. This acidity is one of the important parameters that contributes to honey’s antibacterial activity. Certain acids, specifically gluconic acid (approximately 0.5% *w*/*v*), contribute to the acidic nature of honey [26,27,28]. However, this acidity might not be sufficient in diluted honey to exert bactericidal action against many bacteria [24].

### 2.4. Hydrogen Peroxide

**Figure 2:**
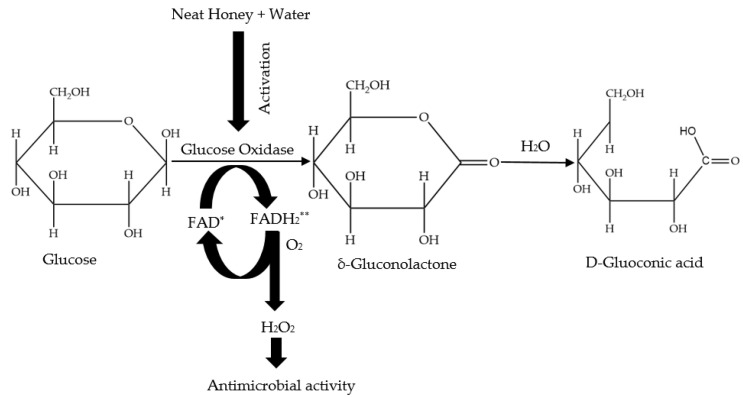
Generation of hydrogen peroxide through the calalyzed reaction by glucose oxidase.

Hydrogen peroxide (H_2_O_2_), a potent oxidizing agent is one of the important parameters responsible for the antibacterial activity of honey [29]. The enzyme glucose oxidase is naturally present in an inactive state in honey due to honey’s low water availability and acidity. However, when honey is diluted, glucose oxidase is activated and takes action on glucose to produce H_2_O_2_ (Figure 2); the maximum level of H_2_O_2_ produced, potentially ranging between 5 and 100 µg H_2_O_2_/g honey [26], can be obtained from a 30–50% honey dilution [24]. According to Bang et al. [30], the production of H_2_O_2_ in some honeys can rise constantly over time to a maximum point subject to the dilution used. H_2_O_2_ levels in honey might reach 2.5 mmol in 30 min and this can increase twofold on extended incubation. Scholars have measured the level of H_2_O_2_ in a large number of honeys and found the average value to be 1 mM [30,31,32,33,34,35]. Interestingly, H_2_O_2_ levels of between 1 and 2.5 mM were found to be sufficient to kill *E. coli* in just 15 min [36,37].

In addition, there is an inverse relationship between catalase (an enzyme naturally present in honey) and honey’s glucose oxidase activity. Thus, the interaction of these two enzymes will impact on H_2_O_2_ generation, which is ultimately linked to honey’s antibacterial activity [38,39]. Catalase originates from pollen and to a lesser extent from nectar and its action differs, subject to the botanical origin of honey [39]. Catalase catalyses the breakdown of H_2_O_2_ to water and free oxygen, a reaction in which the H_2_O_2_ efficiently performs as both electron donor and acceptor. Weston [22] demonstrated that the levels of catalase and glucose oxidase and the subsequent antibacterial activity of honey are interrelated. According to Weston, high glucose oxidase levels lead to the generation of high H_2_O_2_ levels as do low levels of catalase. Studies have revealed that the addition of catalase to honey is inadequate to eliminate total antibacterial activity [22,40,41], thus it can be concluded that in these cases the antibacterial activity of honey is not merely due to the activity of glucose oxidase and the generated H_2_O_2_.

### 2.5. Non-Peroxide Antibacterial Compounds

**Figure 3:**
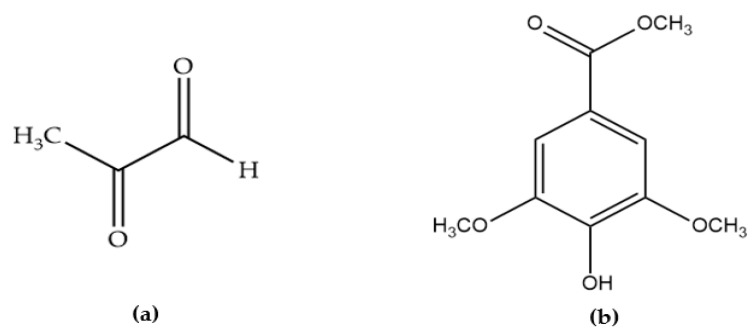
Structure of (**a**) methylglyoxal (MGO) and (**b**) methyl syringate (MSYR).

Several so-called non-peroxide features have been identified as contributing to the antibacterial activity of honey [42]. As mentioned previously, honey may keep its antibacterial action even in the presence of catalase (thus in the absence of hydrogen peroxide). Consequently, these honeys are referred to as “non-peroxide honeys” [42,43]. According to some studies, honey contains simple phenolic and flavonoid compounds, which might play a part in its antibacterial activity [25]. Several other components are known to also contribute significantly to honey’s non-peroxide activity, for example, the occurrence of methyl syringate (MSYR) (Figure 3) and methylglyoxal (MGO) (Figure 3), which have been comprehensively investigated in Manuka honey harvested from the Manuka tree (*Leptospermum scoparium*) [23,44]. MGO is produced from the precursor molecule dihydroxyacetone (DHA), which exists at high levels in the pollen and nectar that bees collect from various *Leptospermum* species [25]. A good direct relationship has been found between MGO content and the antibacterial activity of Manuka honey [43]. Manuka honey can contain up to about 800 mg/kg MGO, which is roughly 100 times more than what is usually found in non-Manuka honey [23,44,45]. This demonstrates that the highly appreciated antibacterial activity of Manuka honey may be associated with its MGO content [45]. As MGO is a honey artefact formed from its nectar derived precursor molecule DHA, the concentration of MGO increases as Manuka honey matures in the hive and also during storage after its harvest. This conversion is temperature sensitive and seems to be optimal in warm but not hot environments (about 37 °C) [44].

Bee defensin-1, also called royalisin is a newly recognized antimicrobial peptide found in Revamil^®^ source (RS) honey [46], which is produced in a standardized greenhouse environment. This peptide was previously already identified in a few other bee related sources such as in honeybee haemolymph, in honeybee heads, in bees’ thoracic glands and also in royal jelly [47,48,49]. Bee defensin-1 has been shown to have strong antibacterial activity but only against Gram-positive bacteria including *B. subtilis*, *S. aureus* and *Paenibacillus larvae* [46,50,51]. Even though bee defensin-1 is readily measureable in RS honey, it is has not yet been found in Manuka honey [52].

Considering the briefly reviewed extensive variety of features that contribute in different ways to the antibacterial activity of honey, it is a difficult undertaking to adequately assess the antibacterial properties of neat honey and by extension, honey-based medicinal formulations. For example, some effects, such as H_2_O_2_ activity, are dependent on the presence of an adequate volume of water (or wound fluid), whereas for others (e.g., acidity, osmolarity), the dilution of honey might negatively impact on their antibacterial effect. Moreover, honeys of different botanical origins might exert different levels of antibacterial activity based on the presence of very different bioactive molecules (e.g., peroxide vs. non-peroxide honeys, MGO vs. bee defensin-1). To establish an assay that adequately captures these different influences and also allows for a meaningful inference of in vitro findings to a clinical setting is therefore a difficult undertaking. As a starting point, the following section provides a brief review of the various methods currently used to determine the antibacterial activity of neat honey as well as their advantages and disadvantages. The discussion then moves on to techniques that are currently in use to test the antibacterial effect of medicinal formulations in order to assess their potential suitability for the testing of honey based preparations.

## 3. In Vitro Assessment of the Antibacterial Activity of Honey

The antibacterial property of honey has been appreciated for hundreds of years, even without accurate knowledge of its precise mechanisms of action. Van Ketel [53] first explained the antibacterial activity of honey through the term ‘inhibine’. At that time, ‘inhibine’ was identified as H_2_O_2_ and determined to be the key antibacterial component in honey [26]. Numerous approaches have since been employed to assess the antibacterial activity of honey, for example, the broth micro-dilution assay, the well/disc diffusion assay, the phenol equivalence assay and also the time-kill assay (Table 1). These techniques are frequently utilized in microbiological fields and many, but not all, are conducted in line with the Clinical and Laboratory Standards Institute (CLSI) guidelines.

### 3.1. Agar Diffusion Assay

**Figure 4:**
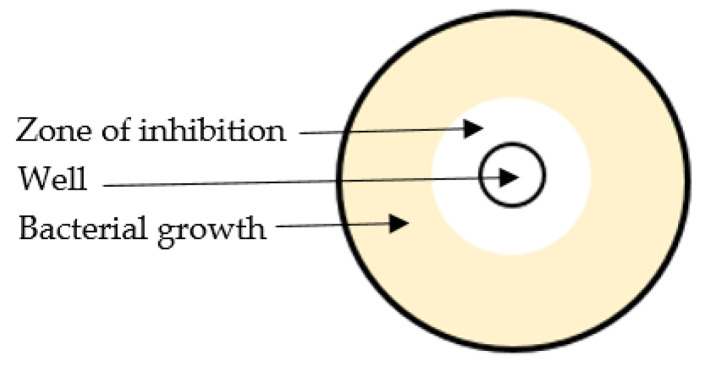
Schematic representation of agar diffusion method.

The agar well diffusion assay is extensively applied to assess the antimicrobial activity of natural products [83,84]. This assay is based on the measurement of the size of a growth inhibition zone around the sample, which may be placed onto a paper disc or into a well cut into the agar. Briefly, in this assay the agar plate surface is inoculated by spreading a volume of the microbial inoculum over the entire agar surface. Then a circular hole (6–8 mm) is made aseptically into the agar using a sterile cork borer, and a suitable volume (20–100 µL) of the sample solution at the chosen concentration is applied into the well. The agar plate is then incubated under proper conditions to allow the antibacterial agent to diffuse into the agar medium and inhibit the growth of the microbial strain tested. This results in a measurable growth inhibition zone (Figure 4).

While the agar diffusion assay is simple and fast to perform, in the context of honey analysis it is hampered by some shortcomings [51,85]:

- The high viscosity of honey creates difficulties with the loading of a definite volume of the sample into the agar wells. This is particularly challenging when the honey is crystallised;
- The diffusion of high molecular weight active constituents (e.g., bee defensin-1) into the agar matrix might be hindered. As a result, the obtained diameter of growth inhibition zones might be comparatively low and not necessarily reflective of the honey’s overall antimicrobial effect;
- The assay tends to have a low discriminatory power when relatively small growth inhibition zones are detected;
- The obtained results may have relatively low levels of reproducibility, which makes inter-lab comparisons of generated data difficult.

The problem of honey’s high viscosity can be, at least partially, overcome by using honey dissolved in sterile water (e.g., 50%, w/w), as it has been suggested by some authors [86,87], but other challenges as listed above remain and these therefore limit the use of the agar diffusion assay for the assessment of neat honey’s antibacterial activity.

### 3.2. Agar Disc Diffusion Method

**Figure 5:**
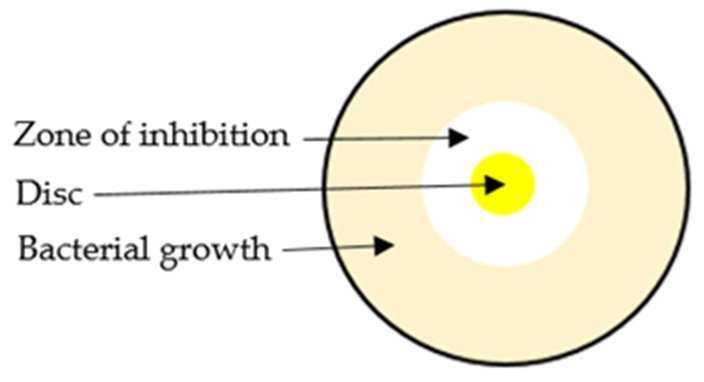
Schematic representation of disc diffusion method.

Agar disc-diffusion testing, developed in 1940, is the approved technique applied in various clinical microbiology laboratories for regular antimicrobial susceptibility testing [86]. Though not all critical bacteria can be tested precisely by this method, the standardisation permits for the testing of a broad range of critical bacterial pathogens like *Streptococci*, *Haemophilus* and *Neisseria* [87,88]. The benefits of this method, mostly its straightforwardness and cost-effectiveness, have contributed to its widespread application for the investigation of antimicrobial testing of plant extracts, essential oils and drugs [89,90,91]. Briefly, agar plates are inoculated with a standardised inoculum of the test microorganism. Next, filter paper discs (approximately 6 mm in diameter), holding the sample solution at a specific concentration, are positioned on the agar surface and the petri dishes are incubated under proper conditions required for the growth of the tested microorganism. The antimicrobial agent disperses into the agar and inhibits the growth of the test microorganism creating a zone of growth inhibition surrounding the sample-impregnated disc (Figure 5).

There are several limitations to the disc diffusion assay as listed below [92]:

- Alike the agar well diffusion assay, the disc diffusion method is unable to capture the antibacterial activity of honey compounds with low ability to diffuse into the agar matrix (e.g., high molecular weight compound such as bee defensin-1). Consequently, the diameters of detected growth inhibition zones might be comparatively small, non-discriminatory, and may not be necessarily reflective of the honey’s overall antimicrobial effect;
- Additionally, the agar disc diffusion technique is not suitable to assess the minimum inhibitory concentration (MIC).

### 3.3. Broth Dilution Method

Broth micro- or macro-dilution techniques are one of the most fundamental and important antimicrobial susceptibility testing methods for natural products including honey against anaerobic and aerobic bacteria [93]. Unlike the aforementioned methods, the broth dilution method allows quantitative investigation of both bacteriostatic and bactericidal activity. Bacteriostatic activity is characterised by the sample’s Minimal Inhibitory Concentration (MIC), the lowest concentration that inhibits the growth of the tested bacterium, while bactericidal activity is characterised by the samples’ Minimal Bactericidal Concentration (MBC), the lowest concentration needed to kill a particular bacterium [92]. There are also numerous other benefits of such a serial dilution method in comparison with the previously mentioned agar diffusion assay, for example more reproducible results, which are also easier to interpret [87].

**Figure 6:**
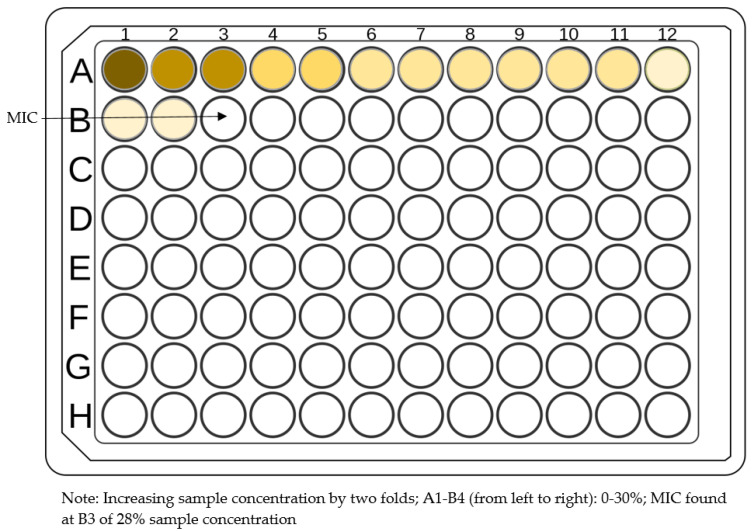
Schematic representation of broth dilution method.

Briefly, for a microbroth dilution assay, specifically in the context of honey testing, inocula are arranged by culturing strains on blood agar overnight at 37 °C, then suspending colonies in 0.85% saline. Different honey concentrations (0–30%) are placed into microtitre plates and in each well a specific cell suspension is added. The microtitre plates are incubated statically for 24 h at 37 °C, after which MICs are measured visually as the lowest concentration of honey resultant in an optically clear well (Figure 6) [58].

A minor modification of the serial dilution method can be also employed for the assessment of the anti-biofilm activity of honey [59]. The minimum biofilm eradication concentration (MBEC) of honey, which is the lowest concentration of a sample needed to eradicate biofilm formed by a particular bacterium, can be determined by this method. Overall, the assay is carried out in the same way as was described for the determination of MIC or MBC values although here the bacterial biofilm is first grown in the wells of the titration plates and then the biofilm is quantified by crystal violet staining rather than visual assessment [59].

Although offering some advantages over other approaches, the microbroth dilution method is not without limitations. The challenging step of this method is preparing accurate honey solutions as the weighing of honey might be challenging due to its high viscosity.

### 3.4. Phenol Equivalence Assay

The phenol equivalence (PE) assay is a variation of the agar well diffusion assay and currently the industry-adopted method used to measure the antibacterial activity of honeys in Australia and New Zealand. In the PE assay, activity is determined by measuring the zone of growth inhibition and stated as equivalence to dilutions of phenol [94,95].

**Figure 7:**
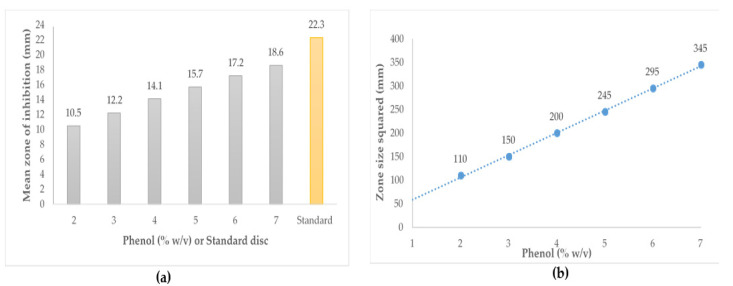
Schematic representation of (**a**) zone sizes for phenol standards and antibiotic disc (**b**) phenol standard curve.

To conduct the PE assay, an 18 h culture of *Staphylococcus aureus* ATCC 25923 in tryptone soy broth (TSB) is prepared (approx. 5 × 10^7^ cells/mL). Next, 150 mL of nutrient agar is seeded with 100 µL of the prepared bacterial culture and poured into a large square bioassay plate. Diluted honey samples are then filter sterilised through 0.2 µm pore filters and mixed with equal volumes of sterile deionised water to make 25% (w/v) concentration. Aliquots of 100 µL of each solution are placed into prepared wells (8 mm) of the assay plates. Phenol standards (2, 3, 4, 5, 6, 7) are prepared from a 10% *w/v* solution that is freshly prepared. Aliquots of 100 µL of each phenol dilution are placed in duplicate wells of the assay plates. ‘Artificial honey’, sterile deionised water and catalase solution are considered as negative controls. The plates are incubated at 37 °C for 18 h. After incubation, all zones of inhibition are measured to the nearest millimetre by eye using a ruler. Each zone is measured at least twice in different directions (preferably at right angles) to confirm that well diameter measurements are representative. A linear standard curve is generated from the mean squared diameter of zone sizes for phenol solutions. The activity of each honey sample is then calculated using the equation obtained from the phenol standard curve (Figure 7) [58,94].

The PE assay is a modified form of the agar well diffusion method and is hampered by similar limitations. The assay relies on diffusion of active components into the agar matrix, which may be problematic for some active components present in honey (e.g., high molecular weight compound such as bee defensin-1). Moreover, this assay has low discriminatory power because it is unable to differentiate between honeys of relatively low antibacterial activity and also not between honeys of very high activity [58].

### 3.5. Time-kill Assay (Time-kill Curve)

The time-kill test is one of the suitable methods for investigating bactericidal or fungicidal effects. It is an appropriate technique for obtaining information about the dynamic interaction between the antimicrobial agent and the respective microbial strain as it reveals information about a time-dependent or a concentration-dependent effect [81]. This method can be used to analyse the synergism or antagonism between two or more agents when tested in combination [81,96]. Moreover, some antifungal agents have been evaluated by this method [97,98].

**Figure 8:**
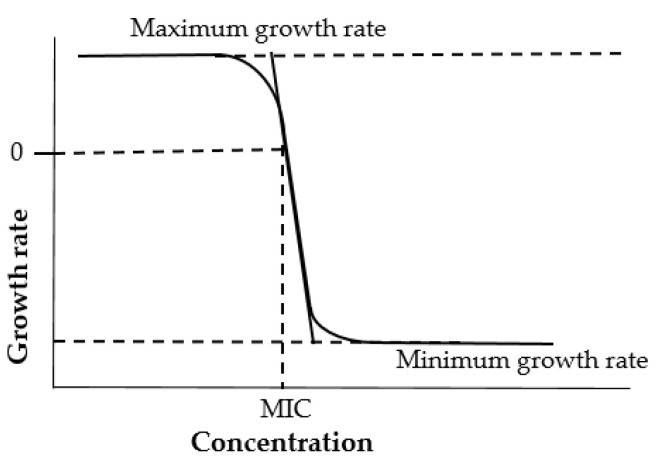
Schematic representation of time-kill curve.

For the time-kill assay in the specific context of honey, inocula of bacteria are arranged by culturing organisms overnight in Tryptic Soy Broth at 37 °C and diluted (about 5 × 10^8^ CFU/mL) in 0.85% saline. Honey solutions (50% *w/v* in 0.85% saline) are inoculated with test organisms ensuring in a final honey concentration of 40% (w/v) and inoculum concentration of about 2.5 × 10^6^ CFU/mL. 0.85% Saline solution is also inoculated in the same way and used as negative control. Inoculated honey solutions and controls are incubated at 37 °C with shaking. Samples are removed at several time points (0, 1, 2, 3, 4, 6, 12 and 24 h) and viable counts are obtained by serial dilution of samples tenfold in 0.85% saline and spot inoculating 10 μL volumes in duplicate from the respective dilutions onto nutrient agar. Agar plates are incubated at 37 °C for 18 to 24 h, and the surviving organisms are counted (Figure 8) [82,89].

Although the time-kill assay allows for the monitoring of bacterial growth and death over a wide range of sample concentrations to evaluate the antimicrobial effect over time, it has some limitations. The assay is very laborious and the prompt bactericidal effects of few agents can result in bacterial counts below the limit of detection (2 × 10^3^ CFU/mL) at the first time point [99,100].

### 3.6. Bacterial Overlay Assay

**Figure 9:**
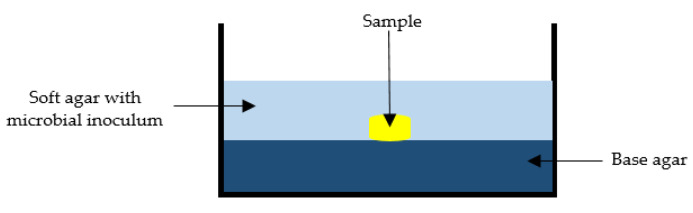
Schematic representation of bacterial overlay assay.

The overlay assay was developed by Gratia in 1936 in the context of counting bacteriophages [101]. The technique has been extensively used in many areas of microbiological research [102]. In short, it allows for the production of a homogeneous lawn of bacteria, which are seeded in soft agar and overlaid onto the base agar with the sample already in place (Figure 9). Soft agar contains a lower concentration of agar and thus allows active constituents in the samples to diffuse more easily, addressing some of the limitations discussed earlier in the context of the agar diffusion assays. The key advantages of this method are that it is easy and simple, requires minimal resources and is able to retain the sample’s integrity [102].

The bacterial overlay method was adapted by Kwakman et al. [52] to assess the antibacterial activity of bee defensin-1 isolated from honey. It provided an advantages avenue because, as mentioned before, the antibacterial activity of high molecular weight honey constituents (i.e., bee defensin-1) is difficult to detect in the traditional agar diffusion assays. The bacterial overlay assay has to date not yet been reported in the literature for the assessment of the antimicrobial activity of honey. This is somewhat surprising as the overlay assay does not employ dilute honey solutions and thus might be able to capture those antibacterial elements in honey (e.g., pH, osmolarity, low water activity) that are inherent only to neat honey. The assay also has potential for the testing of the antimicrobial activity of topical medicinal formulations as it allows the intact formulations to be used in the overlay and therefore to be assessed without further processing. Kemme et al. [103], for instance, quantitatively determined the antimicrobial activity of poly (lactic-co-glycolic acid) films loaded with 4-hexylresorcinol (4-HR) using the overlay antibacterial assay.

While their work was focused on a medicinal formulation with a single active ingredient, it indicates that the assay might provide a suitable and cost-effective template for the analysis of natural-product based topical formulations, including those impregnated with honey.

## 4. Conclusions

In summary, honey is unique when compared to other natural products with respect to its physicochemical properties and health benefits. The antimicrobial activity is one of the predominant bioactivities of honey that has been explored in depth. The level of antimicrobial activity varies from honey to honey and is strongly correlated to its floral source, geographical origin and processing techniques. It is established in the literature that an interplay of different parameters, namely low water content, high sugar content, acidity, hydrogen peroxide and non-peroxide compounds, impact on the observed antimicrobial activity of honey. With growing interest in honey’s potential medicinal effects, linked in particular to its antimicrobial and antioxidant bioactivities, honey has started to be incorporated into various medicinal formulation in lieu of using neat honey.

However, current available methods for the assessment of antimicrobial activity of bioactive agents have limitations when applied to honey. Honey’s distinctive physicochemical characteristics (i.e., its high viscosity) make performing some of these assays challenging. Furthermore, methods that rely on the diffusion of active components in an agar matrix may underestimate the antibacterial activity of honeys as it contains a multitude of bioactive constituents, including high molecular weight components that might only poorly diffuse into the agar. Considering the rising popularity of honey based formulations (e.g., hydrogels, dressings, ointments, pastes), it is also necessary to determine the antimicrobial potential of these formulations.

Of the different methods currently applied to the antibacterial assessment of honey, the bacterial overlay assay might be a suitable approach to determine the antimicrobial activity of both neat honey and also honey-based formulations. However, limited information is currently available on this assay, necessitating further research to optimise the approach specifically for use in the analysis of honey and honey-based formulations.

## Figures and Tables

**Table 1:** Methodologies used for the determination of antimicrobial activity of honey and honey products.

Honey/Honey Product	Method	Organism	Reference
Canadian honeys	Broth dilution method	Bacteria	[38]
Revamil^®^ source (RS) honey; Manuka honey (*Leptospermum* spp.)	Broth dilution method; Bacterial overlay assay	Bacteria	[52]
Tea-tree honey (*Leptospermum lanigerum* *Leptospermum scoparium*)	Broth dilution method	Bacteria	[54]
Jelly bush honey (*Leptospermum polygalifolium*)	Broth dilution method	Bacteria	[54]
Super Manuka honey (*Leptospermum polygalifolium*)	Broth dilution method	Bacteria	[54]
Agastache honey (*Agastache rugosa*)	Broth dilution method	Bacteria	[54]
Capillano^®^ honey	Broth dilution method	Bacteria	[55]
Pasture honey	Broth dilution method	Bacteria	[56]
Manuka honey (*Leptospermum* spp.)	Agar disc diffusion method; Broth dilution method	Bacteria	[54,56,57]
Manuka honey (*Leptospermum* spp.)	Broth dilution method	Bacteria	[54,55,56,57,58,59]
Manuka honey (*Leptospermum* spp.)	Phenol equivalence assay; Broth dilution method	Bacteria	[58]
Jarrah honey (*Eucalyptus marginata*)	Phenol equivalence assay; Broth dilution method	Bacteria	[58]
Marri honey (*Corymbia calophylla*)	Phenol equivalence assay; Broth dilution method	Bacteria	[58,59]
Jarrah honey (*Eucalyptus marginata*)	Broth dilution method	Bacteria	[54,59]
Manuka (*Leptospermum* spp.)	Agar disc diffusion method; Broth dilution method	Bacteria	[60]
Tualang honey (*Apis dorsata*) Acacia honey (*Acacia* spp.) Hannon honey	Agar disc diffusion method, Agar diffusion assay; Broth dilution method	Bacteria	[61]
Acacia honey (*Acacia mangium*)	Agar diffusion assay; Broth dilution method	Bacteria	[62]
Gelam honey (*Melaleuca cajuputi*)	Agar diffusion assay; Broth dilution method	Bacteria	[62]
Kelulut honey (*Trigona* spp.)	Agar diffusion assay; Broth dilution method	Bacteria	[62]
Pineapple honey (*Ananas comosus*)	Agar diffusion assay; Broth dilution method	Bacteria	[62]
Tualang honey (*Apis dorsata*)	Agar diffusion assay; Broth dilution method	Bacteria	[62]
Tualang honey (*Apis dorsata*)	Broth dilution method; Time-kill assay	Bacteria	[63]
Monofloral Cuban honeys	Broth dilution method	Bacteria	[64]
Pincushion honey (*Leucospermum cordifolium*)	Broth dilution method	Bacteria; Yeast	[65]
Fynbos honey (*Erica* spp.)	Broth dilution method	Bacteria; Yeast	[65]
Fynbos honey (*Eucalyptus cladocalyx*)	Broth dilution method	Bacteria; Yeast	[65]
Multi-floral Cameroonian honeys	Agar diffusion assay; Broth dilution method	Bacteria	[66]
Slovak blossom honeys	Broth dilution method	Bacteria	[67]
Ukrainian honeys	Broth dilution method	Bacteria	[68]
Surgihoney	Phenol equivalence assay	Bacteria	[69]
Stingless honeybees honey	Agar diffusion assay; Broth dilution method	Bacteria	[70]
*Apis mellifera* white honey	Agar diffusion assay; Broth dilution method	Bacteria	[70]
*Apis mellifera* yellow honey	Agar diffusion assay; Broth dilution method	Bacteria	[70]
Greek Honeys	Agar diffusion assay; Broth dilution method	Bacteria	[71]
Pakistani unifloral honeys	Agar diffusion assay; Phenol equivalence assay	Bacteria	[72]
Saudi honeys	Agar diffusion assay; Broth dilution method	Bacteria	[73]
Pine honey	Broth dilution method	Bacteria	[74]
Bee pollens	Agar diffusion assay	Bacteria; Fungi	[75]
*Apis mellifera* honey	Agar diffusion assay	Bacteria	[76]
Propolis	Broth dilution method	Bacteria; Yeast	[77]
WA Manuka honey (*Leptospermum* spp.)	Agar diffusion assay; Broth dilution method	Bacteria	[78]
Ulmo 90 honey	Agar diffusion assay; Broth dilution method	Bacteria	[79]
Romanian honey; Propolis	Agar diffusion assay; Broth dilution method	Bacteria; Yeast	[80]
Spotted gum honey (*Eucalyptus maculata*)	Broth dilution method; Phenol equivalence assay	Bacteria; Yeast	[81]
Red stringy bark honey (*Eucalyptus macrorrhyncha*)	Broth dilution method; Phenol equivalence assay	Bacteria; Yeast	[81]
Yellow box honey (*Eucalyptus melliodora*)	Broth dilution method; Phenol equivalence assay	Bacteria; Yeast	[81]
Multiple WA honeys	Agar diffusion assay; Broth dilution method; Time-kill assay; Phenol equivalence assay	Bacteria	[82]

## Data Availability

Not applicable.
